# Role of Genetic Testing in Kidney Stone Disease: A Narrative Review

**DOI:** 10.1007/s11934-024-01225-5

**Published:** 2024-08-03

**Authors:** Robert Geraghty, Catherine Lovegrove, Sarah Howles, John A. Sayer

**Affiliations:** 1 Biosciences Institute, Newcastle University, Newcastle upon Tyne, UK; 2 Department of Urology, The Newcastle upon Tyne NHS Foundation Trust, Newcastle upon Tyne, UK; 3 Nuffield Department of Surgical Sciences, University of Oxford, Oxford, UK; 4 Department of Urology, Oxford University Hospitals NHS Foundation Trust, Oxford, UK; 5 Renal Services, The Newcastle upon Tyne NHS Foundation Trust, Newcastle upon Tyne, UK

**Keywords:** Kidney stone disease, Calculi, Urolithiasis, Nephrolithiasis, Genetics, Genomics, Next generation sequencing

## Abstract

**Purpose of Review:**

Kidney stone disease (KSD) is a common and potentially life-threatening condition, and half of patients experience a repeat kidney stone episode within 5–10 years. Despite the ~50% estimate heritability of KSD, international guidelines have not kept up with the pace of discovery of genetic causes of KSD. The European Association of Urology guidelines lists 7 genetic causes of KSD as ‘high risk’.

**Recent Findings:**

There are currently 46 known monogenic (single gene) causes of kidney stone disease, with evidence of association in a further 23 genes. There is also evidence for polygenic risk of developing KSD. Evidence is lacking for recurrent disease, and only one genome wide association study has investigated this phenomenon, identifying two associated genes (*SLC34A1* and *TRPV5*). However, in the absence of other evidence, patients with genetic predisposition to KSD should be treated as ‘high risk’. Further studies are needed to characterize both monogenic and polygenic associations with recurrent disease, to allow for appropriate risk stratification. Durability of test result must be balanced against cost. This would enable retrospective analysis if no genetic cause was found initially.

**Summary:**

We recommend genetic testing using a gene panel for all children, adults < 25 years, and older patients who have factors associated with high risk disease within the context of a wider metabolic evaluation. Those with a genetic predisposition should be managed via a multi-disciplinary team approach including urologists, radiologists, nephrologists, clinical geneticists and chemical pathologists. This will enable appropriate follow-up, counselling and potentially prophylaxis.

## Introduction

Kidney stone disease (KSD) is a painful, often acute condition that has a significant impact on quality of life [1]. Over the last 30 years the prevalence of KSD in the United States of America (USA) has risen to 10% with an incidence of 2%, and approximately half of stone formers will experience a repeat stone episode within 5–10 years [2]. KSD has an estimated heritability of 50% [3], however the pathogenesis of KSD remains incompletely understood, hindering advances in therapeutics to treat and prevent stones. Moreover, we lack effective methods to predict which patients will experience disease recurrence. Thus, at present, KSD is largely managed surgically with a significant economic burden on health systems [4, 5]. It is estimated that by 2030 the annual cost of treating kidney stones will be nearly $4 billion in the USA (adjusted for 2024 prices) [5].

American and European urological guidelines recommend that “high risk” stone formers should be considered for specific metabolic testing [6, 7]. This includes paediatric cases, patients with a family history of KSD, those suffering from diseases associated with stone formation (such as disorders of mineral metabolism, kidney, or gastrointestinal diseases), patients with anatomical abnormalities, and patients with genetic abnormalities linked to KSD [6, 7]. The evidence for genetic associations with KSD has been building over the past two decades with insights from studies of individual genes [see Table 1] and genome-wide association studies (GWAS) in population-based studies and rare disease cohorts [8]. In twin studies, the heritability of KSD (the proportion of variability in a trait that is attributable to variation in genetic factors. [9]) is estimated to be ~45% [10], however, contemporary studies of genetic risk factors for KSD have only identified a SNP-based heritability of ~20% [11]. Whilst KSD is recognised to have a strong underlying genetic basis there is a lack of consensus as to the role of genetic testing in the field. This article will consider our current understanding of the genetic basis underlying KSD, how this can be applied to clinical practice and guidelines, and determine what gaps remain to be filled.

**Table 1:** Table of genes known to cause or associated with conditions leading to kidney stone disease

**Disorder**	**Genes**	**Inheritance Pattern**	**MIM-Phenotype number / Reference**	**Genomics England Rating**	**Phenotype**	**Management**
**Hypercalciuria**						
Familial Hypercalciuria	*ADCY10*^a^	AD	605205	Red	Normocalcaemia, susceptibility to KSD	Thiazide diuretics
	*VDR*^a^	AD	277440	Red		
	*CLDN2*	XLR	301060	No rating		
	*ALPL*	AD/AR	146300	No rating		
Autosomal Dominant Hypocalcaemia	*CASR*^a^	AD	601198	Green	Hypocalcaemia, hyperphosphataemia, inappropriately low PTH	Use caution with vitamin D and hypocalcaemia correction as they might cause hypercalciuria
	*GNA11*	AD	615361	Red		
Bartter Syndrome	*BSND*	AR	602522	Green	Hypokalaemia, metabolic alkalosis	Indomethacin, aldosterone antagonist, electrolytes as required
	*CASR*^a^	AD	601198	Green		
	*CLCN5*^a^	XLR	[67]	Green		
	*CLCNKA*	AR	613090	Red		
	*CLCNKB*^a^	AR/AD	607364	Green		
	*KCNJ1*^a^	AR	600359	Green		
	*SLC12A1*^a^	AR	601678	Green		
Dent disease	*CLCN5*^a^	XLR	300009	Green	Nephrocalcinosis with low molecular weight proteinuria and progressive CKD. With *OCRL* may include intellectual disability and other features of Lowe syndrome	Screen for osteomalacia, phosphate replacement without vitamin D
	*OCRL*^a^	XLR	300555	Green		
Familial hypomagnesaemia	*CLDN16*^a^	AR	248250	Green	Low serum calcium and magnesium; *CLDN19* associated with ocular phenotype	Magnesium replacement, caution with vitamin D replacement, assess for ocular phenotypes with *CLDN19* variants
	*CLDN19*^a^	AR	248190	Green		
Fanconi Syndrome	*HNF4A*^a^	AD	125850	Green	Fanconi syndrome; maturity onset diabetes of the young; KSD	
Hypophosphataemic Rickets	*SLC34A1*^ab^	AR/AD	612286/ 613388	Green	Low serum phosphate, increased phosphate excretion, elevated 1,25-dihydroxyvitamin D	Screen for osteomalacia; phosphate replacement without vitamin D
	*SLC34A3*^a^	AR/AD	241530	Green		
	*SLC9A3R1*^a^	AD	612287	Amber		
	*PHEX*	XLR	307800	Green		
**Hypercalcaemia**						
Familial hypocalciuric hypercalcaemia	*CASR*^a^	AD	145980	Green	Hypercalcaemia, hypo/hypercalciuria. Can develop KSD if hypercalciuria	Benign – no treatment needed. Stop medications inducing hypercalciuria
	*AP2S1*	AD	600740	Red		
Infantile Hypercalcaemia	*CYP24A1*^a^	AR	143880	Green	Hypercalcaemia – see also hypophosphataemic rickets	*CYP24A1*: inhibitor of vitamin D synthesis e.g. Fluconazole; *SLC34A1*: phosphate replacement
	*SLC34A1*^ab^	AR	616963	Green		
**Cystinuria**						
Cystinuria	*SLC3A1*^a^	AR/AD	220100	Green	Solitary Cystinuria	Urinary alkalinization; thiol-binding drugs; aggressive urinary dilution
	*SLC7A9*^a^	AR/AD	220100	Green		
**Hyperuricosuria**						
Defective purine metabolism	*HPRT1*^a^	XLR	300323	Green	Hyperuricaemia, learning disability, renal failure, hearing loss (*PRPS1*)	Urinary alkalinization, allopurinol / febuxostat, hearing screen, monitor renal function
	*PRPS1*	XLR	300661	No rating		
Renal uric acid wasting	*SLC22A12*^a^	AR	220150	Green	Hypouricosuria	Urinary alkalinization; allopurinol or febuxostat
	*SLC2A9*^a^	AR/AD	612076	Green		
**Xanthinuria**						
Xanthinuria	*XDH*	AR	278300	Green	Hypouricosuria, *MOCS1*, *MOCS2* and *GPHN* variants result in increased serum sulfite levels that can cause seizures and hypotonia	Low purine diet; neurological screening for *MOCS1*, *MOCS2* and *GPHN* variants
	*MOCOS*	AR	603592	Green		
	*MOCS1*	AR	252150	No rating		
	*MOCS2*	AR	252160	No rating		
	*GPHN*	AR	615501	No rating		
**Hyperoxaluria**						
Primary hyperoxaluria	*AGXT*^a^	AR	259900	Green	Renal impairment, hyperoxalaemia, systemic oxalosis	Pyridoxine or Lumasiran for *AGXT* mutations. Potassium citrate, magnesium oxide and/or orthophosphate, aggressive urine dilution,
	*GRHPR*^a^	AR	260000	Green		
	*HOGA1*^a^	AR	613616	Green		
	*SLC26A1*^a^	AR	167030	Red		
Enteric Hyperoxaluria	*SLC26A6*	AD	610068	Red	Hyperoxaluria and KSD demonstrated in mice – theoretically similar action in humans	Limit oxalate intake
**Failed Urinary Acidification**						
Renal tubular acidosis	*ATPV0A4*^a^	AR	602722	Green	Hypokalaemia, hyperchloraemia, hypercalciuria, hyperphosphaturia, hypocitraturia. Hearing loss with *ATP6VB1/C2* and *ATP6V0A4.* Osteopetrosis with *CA2*	Sodium bicarbonate or alkaline citrate; hearing screen; assessment for osteomalacia / osteopetrosis
	*ATP6V1B1*^a^	AR	267300	Green		
	*ATP6V1C2*^a^	AR	618070	No rating		
	*CA2*^a^	AR	259730	Green		
	*FOXI1*^a^	AR	600791	No rating		
	*SLC4A1*^a^	AD / AR	179800	Green		
	*WDR72*^a^	AR	613211	Amber		
**Dihydroxyadenine Crystals**						
Adenine Phosphoribosyltransferase deficiency	*APRT*^a^	AR	614723	Green	Radiolucent kidney stones and renal impairment	Allopurinol or Febuxostat

Amalgamated from Genomics England R256 panel [64], Howles and Thakker [68] and Halbritter [69]

*KSD* Kidney stone disease

^a^Feature on Bern Kidney Stone Registry panel [41]

^b^associated with recurrent disease on GWAS [24]

## Terminology

Before exploring the evidence behind the genetic basis of KSD, it is worth (re)visiting terminology utilised in genetic research. We briefly describe the terms used in this review in Box 1.

**Box 1:** Genetic Research Terminology

**Genetic Research Terminology**	
**Mendelian inheritance**	Also known as monogenic. Mutation in single gene causing disease.
**GWAS**	Genome wide association study
**Locus**	Location within the genome
**Genetic variant**	DNA nucleotide sequence (allele) that is divergent from the most common allele
**ACMG criteria**	American College of Medical Genetics guideline on how variants should be assessed and described42
**SNP**	Single nucleotide polymorphism – variations of a single base pair
**CNV**	Copy number variants - variations in the number of copies of a particular sequence of DNA
**Benign**	as per ACMG criteria
**VUS**	variant of unknown significance – usually intermediate statistical pathogenicity score
**Pathogenic**	as per ACMG criteria
**Diagnostic yield**	proportion of patients in a cohort with positive test result i.e. genetic diagnosis considered to explain their disease
**PRS**	Polygenic risk score – score based on GWAS summary statistics – higher score indicates higher number of represented SNPs within GWAS
***Genetic testing types***	
**Curated gene panel**	examines specific genes e.g Genomics England Nephrolithiasis panel64
**Array**	Ascertains genotype by testing at multiple locations
**Sanger sequencing**	also known as chain determination method - classical method of DNA sequencing performed one sequence at a time. Higher accuracies than NGS, but slower and more expensive. Used for small numbers of genes. Current gold standard for sequencing.
**NGS**	Next Generation Sequencing – also known as massively parallel sequencing – broader analysis at reduced cost using array.
**WES**	Whole Exome Sequencing - entire exome (coding region), not intronic/intergenic (non-coding region)
**WGS**	Whole Genome Sequencing - entire genome (coding and non-coding regions)

## Genome Wide Association Studies

The last two decades have seen GWAS conducted with increasing frequency across a range of genetic ancestries with the aim of identifying genomic loci associated with KSD [11–18]. Akin to a case-control study, in a GWAS, researchers analyse millions of genetic variants across the genome and compare individuals with a disease to those without a disease to detect variants that have statistically significant associations with the disease under investigation. GWAS can also be conducted on continuous traits, for example 24-h urine calcium concentrations; however, GWAS of urinary biochemistry are limited by the quality of data collection, which is hampered by variability in trait measurements, adherence to collection protocols, access to concurrent dietary data, and sample size. Whereas the largest published GWAS in KSD comprises almost 18,000 cases and ~720,000 controls and has identified 44 loci associated with KSD [18], the largest published GWAS of 24-h urinary biochemistry comprises ~6,500 participants and failed to identify any variants reaching GWAS-level significance (p < 5 × 10^-8^) [14].

GWAS have provided insights into common (found in > 1% of the population) genetic variants associated with KSD and several are closely related to genes acknowledged to cause monogenic disorders of nephrolithiasis, for example *CYP24A1*, *SLC34A1* (loss-of-function mutations in these genes cause infantile hypercalcaemia type I and II, respectively [19, 20]), and *CASR* (gain-of-function mutations cause autosomal dominant hypocalcemia [21–23]). Whilst only one GWAS, in an Icelandic population, examined patients with recurrent KSD [24]. This demonstrated two exonic SNPs of interest (i.e. in gene coding regions) in *SLC3A1* (encoding a dibasic amino acid transporter)and TRPV5 (encoding a cation channel) which are both likely to have functional consequences on kidney tubular transport. However, neither reached genome wide significance.

Inferring causality in health and disease is a core goal in much scientific healthcare research. However, common genetic variants identified via GWAS are frequently located in non-coding regions of the genome and have small effect sizes; this restricts our ability to understand their implications on protein-coding genes and biological mechanisms. Several genetic epidemiological techniques can be employed to triangulate evidence and move the field towards the goal of elucidating causal relationships that underpin the pathophysiology of disease including exome-wide association studies, Mendelian randomization (MR), and colocalization.

Exome-wide association studies specifically test for associations of rare, protein-coding genetic variants that frequently have large effect sizes. The contributions of several variants in one gene can be “collapsed” together to elucidate gene-based associations [13]. There is one published exome-wide association study of KSD, performed in the 100,000 genomes cohort, that reports a role for predicted-damaging variants in *SLC34A3* (encoding a sodium-phosphate cotransporter) to be associated with KSD cases compared to controls [8]. This suggests that *SLC34A3* variants have an effect of increasing risk of KSD that is less than that of fully-penetrant Mendelian diagnoses, but greater than those of common variants identified from GWAS.

## Investigating Loci: Mendelian Randomisation and Colocalisation

Mendelian Randomisation (MR) is a genetic epidemiological technique that aims to investigate the causal effects of putative risk factors on biopsychosocial outcomes by exploiting the naturally-occurring genetic variation that exists within a general population [25]. Conventional observational studies are subject to limitations that can lead to inappropriate attribution of “causality” to an exposure variable. These include residual confounding or reverse causality [26]. Randomised control trials (RCTs) can overcome several problems hindering observational studies, however not all research questions are amenable to an RCT, for example investigating exposures that may cause harm or outcomes that have a long latency period, for example some malignancies [27–29]. MR is often considered akin to a genetic RCT. This analogy does not fully capture the intricacies involved in inferring causal relationships however it can be useful to conceptualise the method [30]. Briefly, individuals are allocated to a “study arm” based on the association of their genetic variants with an exposure variable. The odds of an outcome occurring in each genetically pre-defined group is calculated and an odds ratio for the effect of the presence of an exposure on the outcome of interest is generated. The technique can also be applied with continuous traits. MR may be conducted using individual, participant-level data, although it is increasingly common to leverage the power of GWAS results and perform “summary statistic” MR. Studies using this approach have revealed the causal effects of higher albumin-adjusted serum calcium and lower serum phosphate on liability to KSD [11]. Further to identifying causal relationships between exposure and outcome variables, drug-target MR explores the utility of putative drug targets prior to developing lengthy and expensive RCTs [31].

Colocalisation is a statistical genetic method that adopts a Bayesian approach, integrating evidence across all genetic variants in a region, to ascertain whether two or more traits share a common causal signal [32, 33]. The analysis can provide a numerical value of the certainty for the presence of a shared genetic signal and can generate a credible set of candidate, causal genetic variants that account for 95% of the colocalization [32, 33]. MR and colocalization have been used to identify causal variants in *DGKD* (encoding a protein which phosphorylates diacylglcerol, a component of the intracellular Calcium-sensing receptor-signaling pathway), *SLC34A1* (encoding a sodium-phosphate cotransporter), and *CYP24A1* (encoding a protein that initiates the degradation of 1,25-dihydroxyvitamin D3) that link serum biochemical measures of calcium and phosphate with KSD and which point to putative therapeutic targets for future drug studies [34].

## Polygenic Risk

Polygenic risk describes the cumulative effect of multiple effect alleles on an individual’s likelihood of developing disease. Polygenic risk scores (PRS) can be derived from GWAS or ExWAS summary statistics (odds ratios for each variant). The more risk alleles for a trait that an individual carries, the higher their PRS, and statistically, the higher their risk of KSD. To date there has only been one PRS developed for KSD based on GWAS summary statistics [35] from the UK Biobank, whose participants have predominantly white European ancestry. This PRS was tested in the Penn Medicine Biobank, which includes individuals with diverse ancestries. They demonstrated that their PRS was predictive of a KSD diagnosis in people of European American ancestry, but not in those of African American [35]. This demonstrates one of issues posed by the eurocentricity of many genetic epidemiological studies and highlights the need for trans-ancestry research to avoid portions of diverse populations being disadvantaged. To date, no PS has been developed for recurrent KSD.

## Current Clinical Evidence For Genetic Testing in Patients with Kidney Stone Disease

**Fig. 1:**
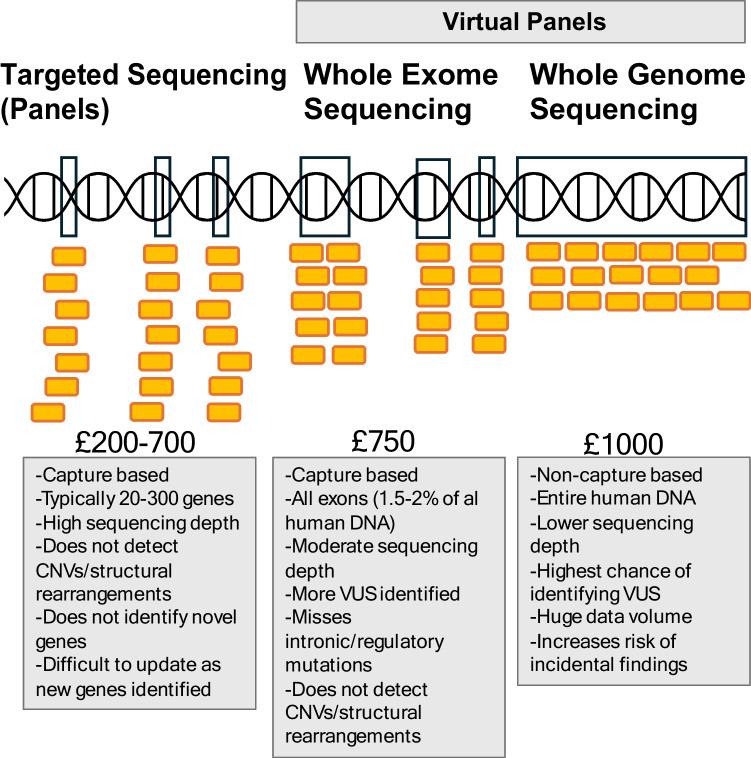
Types of genetic test currently available. Costs from NHS Genomics Education Programme [65]

The current clinical evidence for the role of genetic testing in KSD is comprised of nine cohort studies [see Table 2] [36, 37]. These have been undertaken in children and adults with varying results. Authors have utilised either gene panels or whole exome sequencing (with application of a virtual gene panel) to investigate the diagnostic yields of genetic testing (proportion with a positive genetic diagnosis considered to explain their KSD) in their cohorts [see Fig. 1].

**Table 2:** Summary table of studies examining utility of genetic testing in patients with KSD

**Study**	**Population**	**N**	**Type of Test**	**Diagnostic Variant Definition**	**Diagnostic Yield (%)**
***Children and Young Adults***					
Halbritter et al. [38]	Children with KSD ≤ 18 years – genetically unresolved	106	30 gene panel	PDP, PT, D/LD, HCNH	21%
Braun et al. [39]	Children with KSD ≤ 18 years	123	30 gene panel	PDP, PT, D, HCNH	11%
Daga et al. [70]	Patients with KSD ≤ 25 years	43	WES + virtual 30 gene panel	PDP, D/LD	19%
Cogal et al. [36]	Children with KSD ≤ 18 years with suspected PH or DD	344	90 gene panel	PDP, D/LD, CNV analysis	24% (suspected PH) 7% (suspected DD)
Gefen et al. [43]	Children with KSD ≤ 21 years	83	40 gene panel	PDP, D/LD	12%; 29% when P/LP (D/LD)variants included
Huang et al. [40]	Children with hereditary KSD and extra-renal manifestations ≤ 18 years	19	WES or WGS with CNV analysis	PDP, D/LD, CNV analysis	74%
***Adults***					
Halbritter et al. [38]	Patients with KSD > 18 years	166	30 gene panel	PDP, PT, D/LD, HCNH	11%
Halbritter et al. [38]	Patients with KSD 18–30 years	35	30 gene panel	PDP, PT, D/LD, HCNH	32%
Schönauer et al. [37]	Patients with KSD who had previously undergone intervention > 18 years	236	30 gene panel	PDP, D/LD	1%; 7% when P/LP (D/LD)variants included
Santoro et al. [71]	Patients ≥ 40 years in the INCIPE study (I or II) with a self reported history of KSD	478	Array genome (INCIPE I) or exome (INCIPE II) sequencing	PDP, D/LD	24%
Anderegg et al. [41]	Unselected patients with KSD	787	WES + virtual 34 gene panel	PDP, D/LD	3%; 11% when P/LP (D/LD)variants included

*WES* whole exome sequencing, *KSD* Kidney stone disease, *PH* primary hyperoxaluria, *DD* Dent Disease, *P* Pathogenic, *LP* likely pathogenic, *PDP* previously described as pathogenic, *PT* protein truncating, *HCNH* Healthy controls not homozygous, *CNV* copy number variants, *D* Damaging per Polyphen2 score Halbritter et al. [38] or Polyphen2,SIFT [40] or Polyphen2,SIFT, Mutation Taster Braun et al. [39] & Daga et al. [70, 72, 73] or SIFT, PolyPhen-2 HVAR, MutationTaster, Mutation Assessor, FATHMM, and FATHMM MKL Cogal et al. [36] or Sherloc Gefen et al. [43, 74] or Ensembl VEP, ClinVar Santoro et al. [71] or Ensembl VEP, gnomAD Anderegg et al. [41], *LD* Likely damaging, ascertained as for D [72]

Diagnostic yields seem to be highest in children and young adults. However, there is significant heterogeneity in the criteria for calling a particular variant ‘diagnostic’. Unsurprisingly, when more stringent criteria are utilised, the diagnostic yield diminishes. For example, the studies by Halbritter et al. [38] and Braun et al. [39] were conducted by the same senior authors utilising similar methodologies. Braun et al. applied a more stringent criteria and thus the diagnostic yield in a similar population dropped from 21 to 11%. This yielded a definite diagnostic rate of 12%, with a further 17% having a probable or possible explanatory variant. Whilst in highly selected likely syndromic populations, such as those investigated by Cogal et al. [36] and Huang et al. [40] the diagnostic yield is much higher, although their ‘diagnostic variant’ definitions are less stringent.

In adults, the literature is more varied. In those attending nephrology clinics, usually after referral by urologists, diagnostic yields are higher than in an unselected population. For example, in the adult population studied by Halbritter et al. the diagnostic yield in a selected group of patients attending nephrology clinic was 11% and this increased to 30% on sub-group analysis considering patients aged 18–30 years [38]. In unselected adult cohorts Schönauer et al. [37] and Anderegg et al. [41] the diagnostic yields are reasonable when less stringent criteria are applied (7–11%). However, by limiting analyses to only pathogenic variants (as per ACMG criteria [42]) the yields drop to 1% and 3%, respectively.

To summarise, the genetic diagnostic yields in children and young adults with KSD (≤ 25 years) is between 12–21% [38, 43], whilst in adults the range is 1–11% [38, 41]. These figures should be taken in context, as there is substantial heterogeneity in how diagnostic variants are defined as well as most studies examining selected populations where a genetic diagnosis is suspected. In unselected patient cohorts with stringent diagnostic criteria, only 1–3% of adults carry monogenic variants that are associated with KSD.

## The principles of genetic screening in kidney stone disease

The Wilson & Jungner criteria are an important yardstick to consider when contemplating implementation of a screening test [44, 45]. The need and objectives of genetic screening (at the point of presentation or after) in patients with KSD are relatively clear [see Table 3]; there is a need to identify genetic disease and this information could be leveraged to prognosticate, treat and council the patient and their family. [48, 49] When considering screening a population there are four key considerations: timing (when should a patient be tested), diagnostics (why did the patient develop a stone), prognostics (what will the patient’s disease course be like) and therapeutics (what prophylactic strategies can be utilised). Once a decision is made about whether or not to test, we must then consider which test to use.

**Table 3:** Modified Wilson and Jungner criteria and how this relates to KSD

**Modified Wilson and Jungner criteria** [45]	**Does KSD meet criterium?**
The screening programme should respond to a recognized need	Yes – Prognostication / Treatment
The objectives of screening should be defined at the outset	Yes – Identify Genetic disease
There should be a defined target population	Limited evidence
There should be scientific evidence of screening programme effectiveness	Limited evidence
The programme should integrate education, testing, clinical services and programme management	No evidence
There should be quality assurance, with mechanisms to minimize potential risks of screening	No evidence
The programme should ensure informed choice, confidentiality and respect for autonomy	No evidence
The programme should promote equity and access to screening for the entire target population	Limited evidence
Programme evaluation should be planned from the outset	No evidence
The overall benefits of screening should outweigh the harm	Limited evidence

The time at which a genetic test should be considered is related to the diagnostic yield in the particular population one is testing. Therefore, the time of testing could either be following diagnosis with a first or subsequent stone. There is relatively clear evidence (as detailed above) that the diagnostic yield in patients ≤ 25 years old is 10–20%, and therefore there is reasonable evidence for routine testing in this age group. In unselected adult populations the yields are much lower at ~3%. However, this does rise to ~11% in selected patients attending nephrology clinic. Of particular interest are subanalyses by Schönauer et al. [37] and Anderegg et al. [41]. Schönauer et al. is the only study to investigate factors determining pretest probability of a genetic diagnosis. They identified age at first stone < 40 years, frequent recurrence, mild chronic kidney disease and bilateral KSD [37]. Whilst in Anderegg et al.’s cohort, there were no differences in 24-h urinary biochemistry between those with and those without genetic disease [41]. Together, these suggest that genetic screening could form part of the work up in ‘high risk’ patients > 25 years [41].

Unfortunately, there is incomplete evidence for the natural history of KSD in the context of monogenic causes and therefore we are unable to accurately prognosticate as to the disease course of individual variants. However, the natural assumption is to equate a positive genetic finding with recurrent disease, which is the safe clinical perspective to take until evidence is published to the contrary. Anderegg et al. and Schönauer et al. demonstrated that patients with a genetic diagnosis were significantly more likely to have recurrent disease [41], and have more frequent recurrences [37], respectively. All patients with a likely genetic cause for their disease should be treated as ‘high-risk’.

Finally, therapeutics within the context of KSD represent the most under-researched aspect. Currently there are only a few monogenic disorders that have require specific targetted medications. These include cystinuria (e.g. cystine binding drugs such as penicillamine tiopronin etc. [46]), type 1 primary hyperoxaluria (e.g. pyridoxine (vitamin B6), lumasiran [47] and nedosiran) and infantile hypercalcaemia (e,g, fluconazole [48] and rifampicin [49]). However, treatment strategies within the context of other genetic conditions do not utilise bespoke treatments [see Table 1].

The question then arises of which test to utilise. All the clinical studies have utilised either a gene panel, or a virtual panel applied to WES/WGS data. As our knowledge of causative variants increases, WES and WGS could allow clinicians to retrospectively review initially negative test results to see if a novel variant is represented in a particular patient. However, this is likely not practical within the scope of current practice. Therefore, which test to use remains unclear, and durability of result must be balanced against cost [50]. A gene panel [see Table 1] removes the ability to respectively review test results but is the least expensive. The current clinical evidence is also based solely on gene panels.

## Proposed Diagnostic Algorithm

Current guidance from various sources including the European Association of Urology (EAU), American Urological Association (AUA), Canadian Urological Association (CUA) and National Institute for Health and Care Excellence (NICE) variably suggest that first line investigations should be blood tests of calcium and urate, along with stone analysis. Second line investigations for those that are ‘high risk’ include a variable number of 24 h urine collections to test for volume and electrolytes.

A comprehensive assessment of blood tests to screen for underlying conditions associated with KSD showed that only serum calcium was of clinical utility, that being the diagnosis of hyperparathyroidism. [51] It should also be noted that normocalcaemic hyperparathyroidism has a high prevalence in patients with KSD [52] and therefore PTH should be tested if the patient has high or high-normal serum calcium or normocalcaemic hypercalciuria [53]. However, within the wider context of a full metabolic assessment, other blood tests such as urate become more useful, and therefore should be included. We warn readers not to blindly treat a raised serum urate without a full metabolic assessment.

Twenty four hour urine collections (with or without dietary adjustment) are the current gold standard in detecting biochemical abnormalities associated with underlying causes of KSD. There is growing concern about the clinical utility of these tests [54, 55], difficulties in collection (especially in children) [56] and whether treatments based on these do indeed prevent recurrence [57]. However, until another form of testing is proven to be superior, they remain the gold standard.

**Fig. 2:**
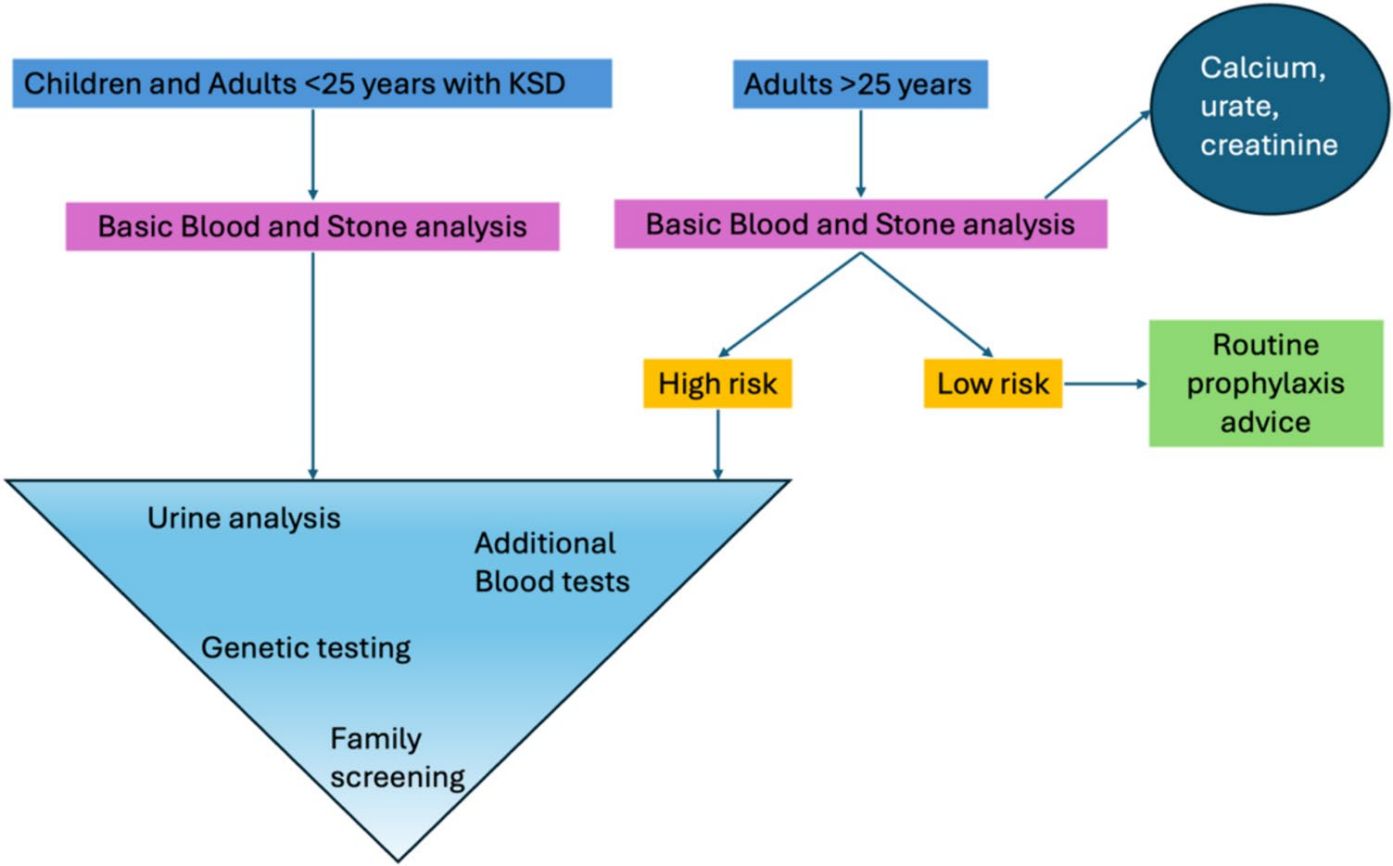
Proposed diagnostic algorithm. High risk as per EAU guidelines. Urine analysis to include ×2 24 h urine collections on random diet to include: calcium, oxalate, phosphate, urate, sodium, creatinine, potassium, magnesium, citrate and volume, with cystine if cystinuria suspected. Urine analysis should also include spot urine for pH and microbiology. Additional blood tests depend upon urine and/or stone analysis results. Hypercalciuria: calcium, alkaline phosphatase, 25 hydroxyvitamin D, 1–25, dihydroxyvitamin D, parathyroid hormone, vitamin A, vitamin D, blood gas analysis, thyroid function tests, bicarbonate. Hyperoxaluria: Plasma oxalate, glycolate, vitamin C. Otherwise: urate, magnesium, blood gas analysis, lactate dehydrogenase, creatinine, urea. Genetic testing in the form of a gene panel, or virtual gene panel applied to WES/WGS depending on institutional availability. If genetic screening demonstrates a likely genetic cause, then family members should be screened. Adapted from Stephan and Hoppe [66]

Given the evidence above, we recommend that genetic testing (in the form of a gene panel testing the genes highlighted in green in Table 1) should be considered in children, adults < 25 years and adults > 25 years if there is clinical suspicion of a metabolic disorder including recurrent (≥ 2 episodes), bilateral disease or strong family history. Genetic testing should only be performed with or after metabolic testing (blood/urine). We have developed a diagnostic algorithm for use in these purposes [see Fig. 2]. We caution readers against ascribing the underlying cause of disease to initial findings e.g. central obesity, as this may be a diagnostic red herring masking an underlying serious diagnosis.

## Future Directions

 Urologists are on the cusp of being able to enhance KSD patient care by leveraging advances made in our understanding of the genetic basis of the disease (as outlined above), however further research is needed to completely define the genetic landscape of KSD and correlate this with phenotypes. By expanding genetic testing, research into this area will accelerate. Genetic testing has several possible applications; the information can be used to counsel patients on the natural history of a disease; to derive more accurate risk stratification tools for a disease; and to inform therapeutic strategies.

As genetic research into KSD becomes more advanced, our understanding of potential intronic causes and polygenic risk should increase. There is increasing evidence implicating genetic variants in non-coding regions of the genome in causing disease, for example cystic fibrosis [58]. Further work is needed to explore this phenomenon within the context of KSD.

At present, unless a rare monogenic disorder such as cystinuria or primary hyperoxaluria is present, there is no accurate prognostic tool for describing the disease course of KSD. Although there are many factors associated with ‘high-risk’ disease [59], prognostic tools developed using these factors, for example the ROKS nomogram, are unable to differentiate between those with and without recurrent disease [60, 61]. Risk stratification, akin to that described for malignancies, needs urgent research to streamline follow-up [62] and allow resource reallocation to those who are likely to experience recurrent disease. Studies to develop accurate prognostication tools require large, robust, and comprehensive datasets, for example longitudinal biobanks. Clinical studies directed at specific phenotypes can be more accurate in terms of the data collected, however they usually lack the scale of biobanks. A combination of the two should be used in the future, for example using clinical studies to validate results derived from biobanks [54–58]. There are complexities surrounding reliably predicting a patient’s risk of recurrent KSD as recurrence is poorly defined, with differing definitions from clinicians [62] and patients [41]. PRS have much lower accuracy than multivariable prediction tools, which is to be expected from what is essentially a single variable [63]. A robust tool incorporating monogenic, polygenic, patient, and environmental data to predict the risk of disease recurrence would be a great advance for patients and endourologists.

A final, and important, aim of research into the genetic basis of KSD is to identify and develop novel therapies to prevent stone recurrence. As the burden of KSD increases, there is increasing interest in prophylactic strategies. However, there is increasing concern that these strategies are not as effective as hoped [57]. This is likely due to poor understanding of the underlying pathophysiology of KSD and treating broad, heterogenous populations in which some patients will respond to therapy and others will not. Increasing understanding of the role of genetic factors in biological mechanisms underpinning KSD presents the opportunity to develop prophylactic treatments and to provide personalised medicine approaches to patients according to their individual risk.

## Conclusion

Research into the genetic basis of kidney stone disease is gaining momentum. There is expanding clinical evidence that gene panels applied to patients ≤ 25 years have reasonable diagnostic yields, whilst those with their first stone aged < 40 years and recurrent or bilateral disease are significantly more likely to have a genetic diagnosis. Any genetic testing must be within the context of a full metabolic screen. However, there are currently very few bespoke treatment strategies for KSD and therefore further work is needed to expand our understanding of the genetics behind KSD and develop novel prophylactic medications. In the future we anticipate that whole genome sequencing and the application of virtual gene panels will become the gold standard as this allows a complete genome landscape analysis as well as retrospective analysis when additional risk alleles are identified in research studies.

## Data Availability

No datasets were generated or analysed during the current study.
